# IVF/ICSI outcomes after culture of human embryos at low oxygen tension: a meta-analysis

**DOI:** 10.1186/1477-7827-9-143

**Published:** 2011-11-01

**Authors:** David B Gomes Sobrinho, Joao Batista A Oliveira, Claudia G Petersen, Ana L Mauri, Liliane FI Silva, Fabiana C Massaro, Ricardo LR Baruffi, Mario Cavagna, José G Franco

**Affiliations:** 1 Department of Gynaecology and Obstetrics, Botucatu Medical School, São Paulo State University - UNESP, Botucatu, Brazil; 2 Center for Human Reproduction Prof Franco Jr, Ribeirao Preto, Brazil; 3 Paulista Centre for Diagnosis, Research and Training, Ribeirao Preto, Brazil; 4 Women's Health Reference Center, Hospital Perola Byington, Sao Paulo, Brazil

**Keywords:** low oxygen, embryo culture, gas atmosphere, IVF/ICSI

## Abstract

**Background:**

Improved pregnancy, implantation, and birth rates have been reported after the use of reduced O2 concentration during embryo culture, mainly due to a reduction of the cumulative detrimental effects of reactive oxygen species. However, some studies have failed to report any positive effects. The objective of this meta-analysis was to evaluate the effect of a low-O2 environment on IVF/intracytoplasmic sperm injection (ICSI) outcomes.

**Methods:**

All available published and ongoing randomised trials that compared the effects of low (~5%; OC~5) and atmospheric (~20%; OC~20) oxygen concentrations on IVF/ICSI outcomes were included. Search strategies included online surveys of databases from 1980 to 2011. The outcomes measured were fertilisation rate, implantation rate and ongoing pregnancy rates. The fixed effects model was used to calculate the odds ratio.

**Results:**

Seven studies were included in this analysis. The pooled fertilisation rate did not differ significantly (*P *= 0.54) between the group of oocytes cultured at low O2 tension and the group at atmospheric O2 tension. Concerning all cycles, the implantation (*P *= 0.06) and ongoing pregnancy (*P *= 0.051) rates were not significantly different between the group receiving transferred sets containing only OC~5 embryos and the group receiving transferred sets with only OC~20 embryos. In a meta-analysis performed for only those trials in which embryos were transferred on day 2/3, implantation (*P *= 0.63) and ongoing pregnancy (*P *= 0.19) rates were not significantly different between the groups. In contrast, when a meta-analysis was performed using only trials in which embryos were transferred on days 5 and 6 (at the blastocyst stage), the group with transferred sets of only OC~5 embryos showed a statistically significantly higher implantation rate (*P *= 0.006) than the group receiving transferred sets with only OC~20 embryos, although the ongoing pregnancy (*P *= 0.19) rates were not significantly different between the groups.

**Conclusions:**

Despite some promising results, it seems too early to conclude that low O2 culture has an effect on IVF outcome. Additional randomised controlled trials are necessary before evidence-based recommendations can be provided. It should be emphasised that the present meta-analysis does not provide any evidence that low oxygen concentration is unnecessary.

## Background

The role of oxygen tension during the culture of gametes and embryos has been the subject of study in both animal models and humans. Following protocols from somatic cell culture techniques, the embryos of humans and other mammals have traditionally been cultured under atmospheric oxygen tension (~20%). However, experimental studies in various species of mammals have revealed that the concentration of O_2 _inside the uterus and oviducts usually fluctuates in the range of 2-8% [1-5]. Culture at low levels of O_2 _(5-7%) can improve embryonic development in several species, including mice [6-10], rats [11], hamsters [12], rabbits [13], pigs [14], goats [15], sheep [16,17], and cattle [16,18]. In addition, culturing at a low O_2 _concentration is associated with a reduced rate of aneuploidy in mouse embryos [19].

In general, these results are associated with a reduction of the harmful effects of reactive oxygen species (ROS). In oocytes and embryos, even with endogenous defence mechanisms [20,21], disturbances in physiological processes can lead to an increase in the generation and accumulation of ROS, which are associated with various degrees of cell damage (DNA fragmentation, changes in gene expression and organelle and membrane disturbances) [7,21-26]. Consequently, interrupted or delayed embryonic development, embryonic fragmentation, apoptosis or health impairment during pregnancy can be observed [21,27,28]. ROS may originate either directly within gametes and embryos (by different enzymatic mechanisms) or from the environment in which they are located [20]. In contrast, the *in vitro *manipulation of gametes in embryos favours the generation of ROS as it involves the exposure of eggs and embryos to xenobiotics, disturbed concentrations of metabolic substrates, traces of transitional elements, light and high oxygen concentrations [20].

On the other hand, the belief that the detrimental effects of atmospheric oxygen tension on embryonic development are caused from the increased production of ROS could be an inexact view of the role of oxygen during embryo development. The culture of early embryos at low O_2 _concentration can influence both cellular mechanisms and gene expression. Induction of the hypoxia-inducible factor transcription family may improve embryonic development and quality following culture at low oxygen levels [29-34].

Assuming that in humans physiological hypoxia also exists in the female genital tract, the results from animal experiments have important implications for the clinical application of IVF/intracytoplasmic sperm injection (ICSI). As reported in animal experiments, beneficial effects of reduced O_2 _levels have also been observed in human studies [28,35-38], including a greater rate of embryonic development up to the blastocyst stage, a faster cleavage rate, an increased blastulation rate, an increase in the number of blastocyst cells and in the number of cryopreserved blastocysts, and an increase in the proportion of high-quality blastocysts. Regarding clinical outcomes, some studies have reported improvements in the implantation rate, pregnancy rate, delivery and live births [21,24,37] with low O_2 _concentrations compared with atmospheric concentrations. Conversely, other randomised studies have failed to yield positive results, reporting no differences in the implantation and pregnancy rates [35,38-40]. Therefore, while several trials have compared the effects of different O_2 _concentrations, definitive conclusions could not be drawn from the individual studies because of conflicting results. Whether the culture of human oocytes and embryos in low concentrations of oxygen can actually improve the clinical results of assisted reproduction cycles is a question that remains to be answered.

Seeking to understand the influence of O_2 _tension, this meta-analysis tests the hypothesis that low O_2 _concentration significantly improves the clinical outcomes compared to atmospheric O_2 _concentration when used for oocyte and embryo culture in infertile patients undergoing treatment with IVF/ICSI.

## Methods

### Criteria for including studies in this meta-analysis

All available published and ongoing randomised controlled trials comparing the effect of low (~5%; OC~5) and atmospheric (~20%; OC~20) oxygen concentrations on IVF/ICSI outcomes were included. Only studies on human IVF/ICSI were included. Frozen embryo replacement cycles were not included.

### Outcome measures

The outcome measures used for this meta-analysis were fertilisation rate, implantation rate and ongoing pregnancy rate (OPR) per transfer.

### Identification of studies

Search strategies included online surveys of the MEDLINE, EMBASE, Science Citation Index, Cochrane Controlled Trials Register and Ovid databases for publications in the years 1980 to 2011. There was no language restriction, and grey literature was included. The following medical subject headings and text words were used: embryo culture, preimplantation development, embryo development, low oxygen, oxygen concentration, oxygen, gas atmosphere, IVF, ICSI, randomised study. The principal inclusion criterion was a randomised controlled trial (RCT).

### Search results

**Figure 1:**
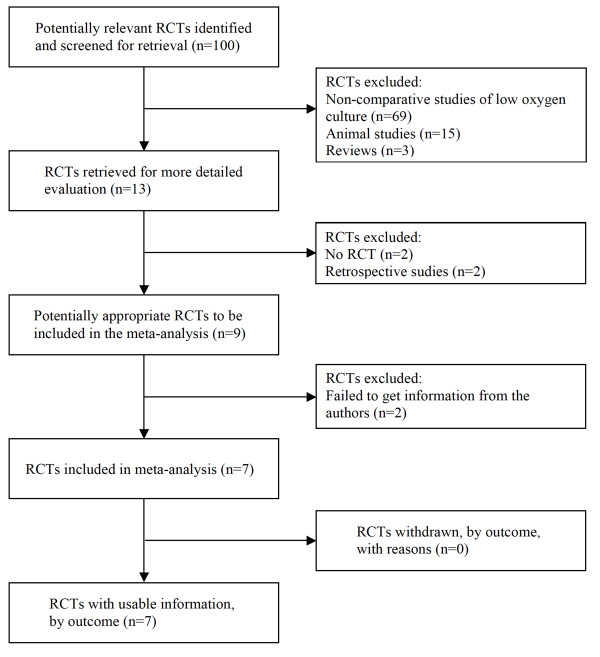
**QUOROM statement flow diagram illustrating the selection of trials included in the meta-analysis**. RCT: randomised controlled trial.

Among the 13 potentially relevant studies retrieved, a total of seven trials fulfilled the inclusion criteria [24,28,35,36,38-40]. A flow diagram of the selection process is shown in Figure 1.

### Validity assessment and data extraction

Each trial was assessed independently by four reviewers (DBGS, JBAO, RB and JGF) and ranked for its methodological rigor and its potential for the introduction of bias. Validity was assessed based on the originally reported characteristics, including a method for randomisation, the presence of a power calculation, the unit of analysis used, and the presence or absence of blinding. Missing data were obtained from the authors when possible.

### Study descriptions - Below are brief descriptions of each study

Dumoulin et al., 1999 [35]: In this prospective randomised study of 1, 380 consecutive IVF treatments, the authors compared the results of culturing human oocytes and embryos for the first 2 or 3 days of development in microdroplets of medium under oil using a gas phase containing either an atmospheric (~20%/690 cycles) or a reduced (5%/690 cycles) O_2 _concentration. No significant differences were found between the two groups cultured under 5% or 20% O_2 _in their rates of fertilisation (60 versus 61%, respectively), embryo development at day 2 or 3, pregnancy (26.6 versus 25.4%, respectively), or implantation (13.4 versus 14.0%, respectively). The culture of surplus embryos under 5% O_2 _resulted in a significantly higher mean incidence of blastocyst formation per cycle compared with the 20% O_2 _group (25.8 ± 2.0 versus 20.4 ± 1.9, respectively). The mean number of embryos classified as blastocysts by microscopic observation of a blastocoele was significantly higher in the 5% O_2 _group than in the 20% O_2 _group, both in blastocysts fixed on day 5 (39.8 ± 1.7 versus 31.9 ± 1.9, respectively) and those fixed on day 6 (45.6 ± 2.6 versus 33.7 ± 3.4, respectively). The authors concluded that although culture under 5% O_2 _led to slightly improved preimplantation embryonic viability, that this effect was either too marginal to result in higher pregnancy rates or that low O_2 _concentrations exerted an effect during the later stages of preimplantation development only.

Bahçeci et al., 2005 [40]: In this prospective randomised study, the authors tested the hypothesis that ICSI outcomes can be improved by culturing human embryos in an atmosphere of controlled O_2 _concentration and compared 5% O_2 _with 20%. From a total of 822 oocyte retrieval cycles, 712 resulted in embryo transfers, of which 255 were transferred at day 2 and 457 were transferred at day 3. Of the 255 day 2 transfers, oocytes and embryos from 118 cycles were incubated in 5% O_2_, and 137 were in 20% O_2_. Of the 457 day 3 transfers, oocytes and embryos from 219 cycles were incubated in 5% O_2 _and 238 in 20% O_2_. The cycle characteristics and the embryology parameters were similar between groups. The embryo qualities were similar with day 2 transfers; however, day 3 transfers incubated in 5% O_2 _were better than those incubated in 20% O_2_. The clinical outcome parameters did not differ between groups with respect to the O_2 _concentration. The authors concluded that culture of embryos under atmospheric O_2 _concentration for the first 2 or 3 days did not alter the clinical outcomes in ICSI cycles.

Kea et al., 2007 [39]: In this study, the effects of two standard oxygen concentrations, low (5% O_2_, 5% CO_2_, and 90% N_2 _/49 patients) and atmospheric (5% CO_2 _with the balance air/57 patients), on fertilisation, embryo development, and pregnancy rate were compared in patients undergoing IVF. No significant differences were noted in the number of patients, patient age, infertility procedures, total number of eggs, total number of 2PNs, fertilisation rate, average number of embryos transferred, and pregnancy outcomes between the groups receiving embryos cultured under the different gas phases. The embryo quality of the day 3 ET groups did show a significant difference between the gas phases (*P = *.0057), implying improved embryo development under low conditions (5% O_2_). However, the embryo quality in the day 5 ET groups showed no significant differences. Only 21% of the patients in this study were eligible for blastocyst transfer. The percentage of patients who had ET on day 5 was 20% in the low O_2 _group and 21% in the atmospheric O_2 _group. Among the patients eligible for blastocyst transfer, there were no significant differences between the gas-phase groups in any of the parameters. The authors concluded that the differences in oxygen concentration did not significantly affect the fertilisation rate, blastocyst formation, or pregnancy rate but that there was a significant difference in the mean embryo scores between the low and atmospheric O_2 _groups on day 3.

Kovacic & Vlaisavljevic, 2008 [28]: Using sibling human oocytes, this prospective study analysed the effects of 5% and 20% oxygen on the prolonged development of embryos. The outcomes measured were fertilisation rate and the proportion of morphologically optimal embryos, blastocysts and optimal blastocysts developed by day 5. The results were analysed separately for the groups on IVF (*n *= 988 oocytes) and ICSI (*n *= 928 oocytes) cycles. It was found that low oxygen did not influence fertilisation, but in comparison with 20% oxygen, it resulted in a significantly higher proportion of embryos being optimal on day 3 after IVF (59 versus 43.2%; *P <*0.001) and after ICSI cycles (51.2 versus 28.5%; *P <*0.001). For both methods, the lower oxygen concentration improved the blastulation rate (73.2 versus 63.1%; *P <*0.05 and 67.4 versus 54.7%; *P <*0.001, respectively) and increased the proportion of embryos reaching the stage of expanded blastocyst with a normal inner cell mass on day 5 (31.1 versus 14.6%; *P <*0.001 and 18.9 versus 11.4%; *P <*0.01, respectively). The ratio of successful embryonic development to optimal blastocyst stage on day 5 of culture calculated for the two oxygen concentrations was 2.1 for IVF and 1.7 for ICSI, clearly favouring lower oxygen tension.

Ciray et al., 2009 [36]: This study was designed to test the hypothesis that low oxygen concentration improved blastocyst yield and quality in human embryo culture. A total of 75 oocyte-retrieval cycles were studied, of which 74 resulted in embryo transfer. This study was conducted with sibling oocytes in which human gametes and embryos were cultured either at low O_2 _conditions (6% CO_2 _+ 5% O_2 _+ 89% N_2 _/868 oocytes) from insemination to day 6 or at atmospheric conditions (6% CO_2 _in air/860 oocytes) until day 3 and at low O_2 _conditions thereafter. All oocytes in the control (atmospheric O_2_) and experimental (5% O_2_) groups were subjected to ICSI. The results showed that low oxygen concentration throughout the culture period improved total blastocyst yield and embryo quality at day 5 and day 3.

Meintjes et al., 2009 [24]: The objective of this study was to evaluate the effect of lowered incubator O_2 _tension on live birth rates in a predominately day 5 embryo transfer program. A total of 230 first-cycle women undergoing routine IVF or ICSI with ejaculated sperm were randomised in a prospective clinical trial and stratified by patient age and physician. The patients' embryos were randomly assigned for culture in either a 21% O_2 _(atmospheric) or a 5% O_2 _(reduced oxygen) environment. The clinical endpoints monitored were the rates of implantation, clinical pregnancy, live birth and blastocyst cryopreservation. The results showed that embryos cultured in a 5% O_2 _environment consistently resulted in higher rates of implantation (106/247, 42.9% versus 82/267, 30.7%; a difference of 12.2% and a 95% confidence interval (CI) of 3.9-20.3, P = 0.005) and live births (66/115, 57.4% versus 49/115, 42.6%; a difference of 14.8% with a 95% CI of 1.9-27.0%, P = 0.043) when compared with rates among women whose embryos were cultured in an atmospheric O_2 _environment. The authors concluded that the overall increase in live births reported by this study indicates that the effort and expense of culturing embryos in a low-O_2 _environment is justified.

Kovacic et al., 2010 [38]: In this prospective randomised trial, the authors aimed to determine whether embryo cultivation at different oxygen tensions had any effect on ICSI outcome. A total of 647 patients were screened for eligibility, and all of them proceeded to randomisation. In 326 patients, the oocytes were assigned to the 5% oxygen treatment (at 6% CO_2_, 5% O_2_, and 89% N_2_), and the oocytes received the 20% oxygen treatment (6% CO_2 _in air) for 321 patients. It was observed that although low oxygen resulted in a higher proportion of quality day 2 embryos and optimal blastocysts, the rates of ongoing pregnancy (31.6% vs. 27.1%) and implantation (28.8% vs. 25.2%) were similar in both treatment groups. However, low oxygen resulted in a higher cumulative pregnancy rate (38% vs. 28.3%) in the main group (all patients) and a higher pregnancy rate in the poor-responder subgroup (23% vs. 9.8%), with embryo transfers performed primarily on day 3. The authors concluded that the use of reduced oxygen in IVF is reasonable, irrespective of the duration of embryo culture.

### Assessment of the risk of bias in the included studies

The method of randomisation was not stated in one of the RCTs [24]. In four studies, serial entry was used, with alternate allocations per set of two treatment cycles [35] according to the day of admission to the embryology laboratory (calendar day: even or odd) [40], by week of oocyte retrieval [39] and alternation (similarly expanded cumulus/MII oocyte) [28]. Procedures for allocation concealment or blinding were not mentioned or were not performed in any of the studies. Co-intervention was observed in one of the trials [35].

The approaches for day 2/3 and day 5 culture were different in the different study centres. One used only short, while others combined short and prolonged cultivation by different criteria for day 2/3 and day 5 transfer. This can cause a bias in the evaluation of success between two groups.

In the included seven studies, research groups used different culture media: P-1 medium+ human serum albumin [24], BlastAssist medium (Medicult, Jyllinge, Denmark) [28,38], house-prepared HTF+ human plasma protein and IVF-50TM (Scandinavian IVF Science AB, Göteborg, Sweden) [35], Quinn's Advantage Plus Cleavage Medium/Blastocyst Medium [36], cleavage media (Sage BioPharma, Trumbull, CT) [39], and G1.3 medium (Vitrolife, Gothenburg, Sweden) [40]. This difference in the composition of the culture medium and the presence of supplements may influence the action of O_2 _tension [41-44].

### Statistical analysis

Data management and analysis were conducted using the StatsDirect statistical software (Cheshire, UK). The fixed effect model was used for the odds ratio (OR), and the effectiveness was evaluated by the Mantel-Haenszel method. A confidence interval (CI) was calculated using the variance formula of Robins, Breslow and Greenland. A chi-squared test statistic was used with its associated probability that the pooled OR was equal to 1. The measure of heterogeneity (non-combinability) was evaluated using Cochran's Q, the Breslow-Day and I^2 ^(inconsistency) tests. A nonsignificant result (i.e., a lack of heterogeneity) indicated that no trial had an OR significantly worse or better than the overall common OR obtained by pooling the data. Because a fixed effects model was employed herein, it is important to acknowledge that inferences refer only to the particular studies included in the analysis. Meta-analysis used in this manner is simply a device to pool the information from the various studies to generate a composite finding, but this finding is only for those studies. Because many of the preceding analyses contained only two or three studies, we decided to derive the inferences from a fixed-effects model.

## Results

### Fertilisation rate (Figure 2)

Six studies were included [24,28,36,38-40]. The pooled fertilisation rates did not differ significantly between the group of oocytes that was cultured at OC~5 (73%, 7, 066/9, 682) and the group cultured at OC~20 (72.5%, 7, 495/10, 342) (*P *= 0.54; OR = 1.02, 95% CI = 0.96-1.09). There was no heterogeneity in this comparison (Breslow-Day = 2.42, df = 5, *P *= 0.79; Cochran's Q = 2.42, df = 5, *P *= 0.79; I^2 ^= 0%, 95% CI = 0% to 61%).

**Figure 2:**
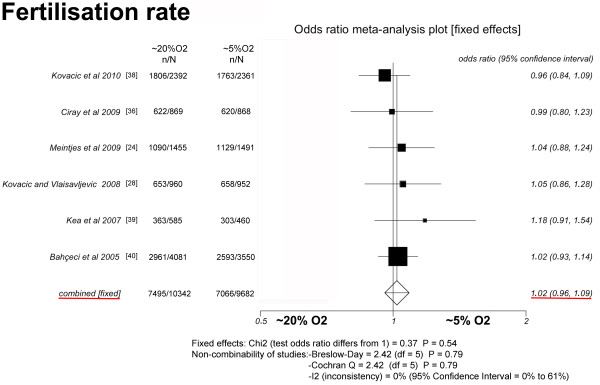
**Fixed-effect model**. Forest plot for fertilisation rate.

### Implantation rate (Figure 3)

Four studies were included [24,35,38,40]. The general implantation rates did not differ between the group of patients that received sets with only OC~5 embryos (22.1%, 768/3, 468) and the group receiving only OC~20 embryos (20.6%, 739/3, 590) (*P *= 0.06; OR = 1.12, 95% CI = 1.00-1.26). There was heterogeneity in this comparison (Breslow-Day = 9.64, df = 3, *P *= 0.02; Cochran's Q = 9.60, df = 3, *P *= 0.02; I^2 ^= 68.8%, 95% CI = 0% to 87.1%).

**Figure 3:**
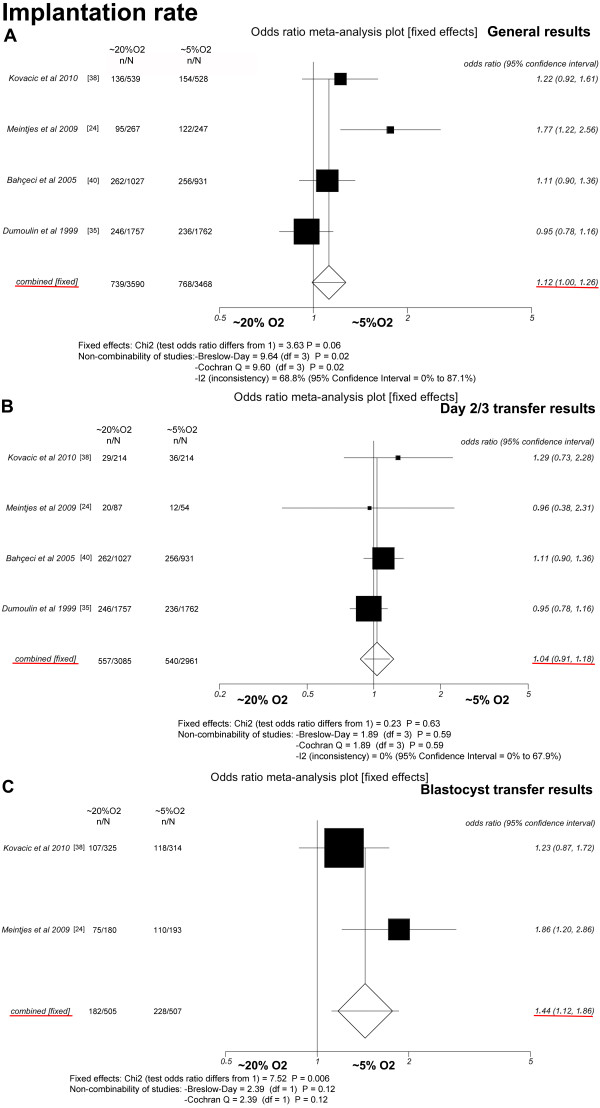
**Fixed-effect model**. Forest plot for implantation rate. A: All cycles; B: only cycles in which embryos were transferred on day 2 or 3; C: only cycles in which embryos were transferred at the blastocyst stage.

Similarly, when a meta-analysis was performed only with cycles in which embryos were transferred on day 2 or 3 [24,35,38,40], the implantation rates were not significantly different between the transferred sets with only OC~5 embryos (18.2%, 540/2, 961) and the sets with only OC~20 embryos (18%, 557/3, 085) (*P *= 0.63; OR = 1.04, 95% CI = 0.91-1.18). There was no heterogeneity in this comparison (Breslow-Day = 1.89, df = 3, *P *= 0.59; Cochran's Q = 1.89, df = 3, *P *= 0.59; I^2 ^= 0%, 95% CI = 0%-67.9%).

However, when a meta-analysis was performed only with cycles in which embryos were transferred at the blastocyst stage [24,38], the implantation rates were significantly different between the transferred sets with only OC~5 embryos (45%, 228/507) and the sets with only OC~20 embryos (36%, 182/505) (*P *= 0.006; OR = 1.44, 95% CI = 1.12-1.86). There was no heterogeneity in this comparison (Breslow-Day = 2.39, df = 1, *P *= 0.12; Cochran's Q = 2.39, df = 1, *P *= 0.12).

### Ongoing pregnancy rate (OPR) per transfer (Figure 4)

Four studies were included [24,35,38,40]. Concerning all cycles, the general OPR per transfer did not differ between the group of patients that received sets with only OC~5 embryos (34.6%, 484/1, 398) and the group receiving sets with only OC~20 embryos (31.6%, 450/1, 423) (*P *= 0.051; OR = 1.18, 95% CI = 1.00-1.38). There was no heterogeneity in this comparison (Breslow-Day = 3.90, df = 3, *P *= 0.27; Cochran's Q = 3.89, df = 3, *P *= 0.27; I^2 ^= 23%, 95% CI = 0% to 74.7%).

**Figure 4:**
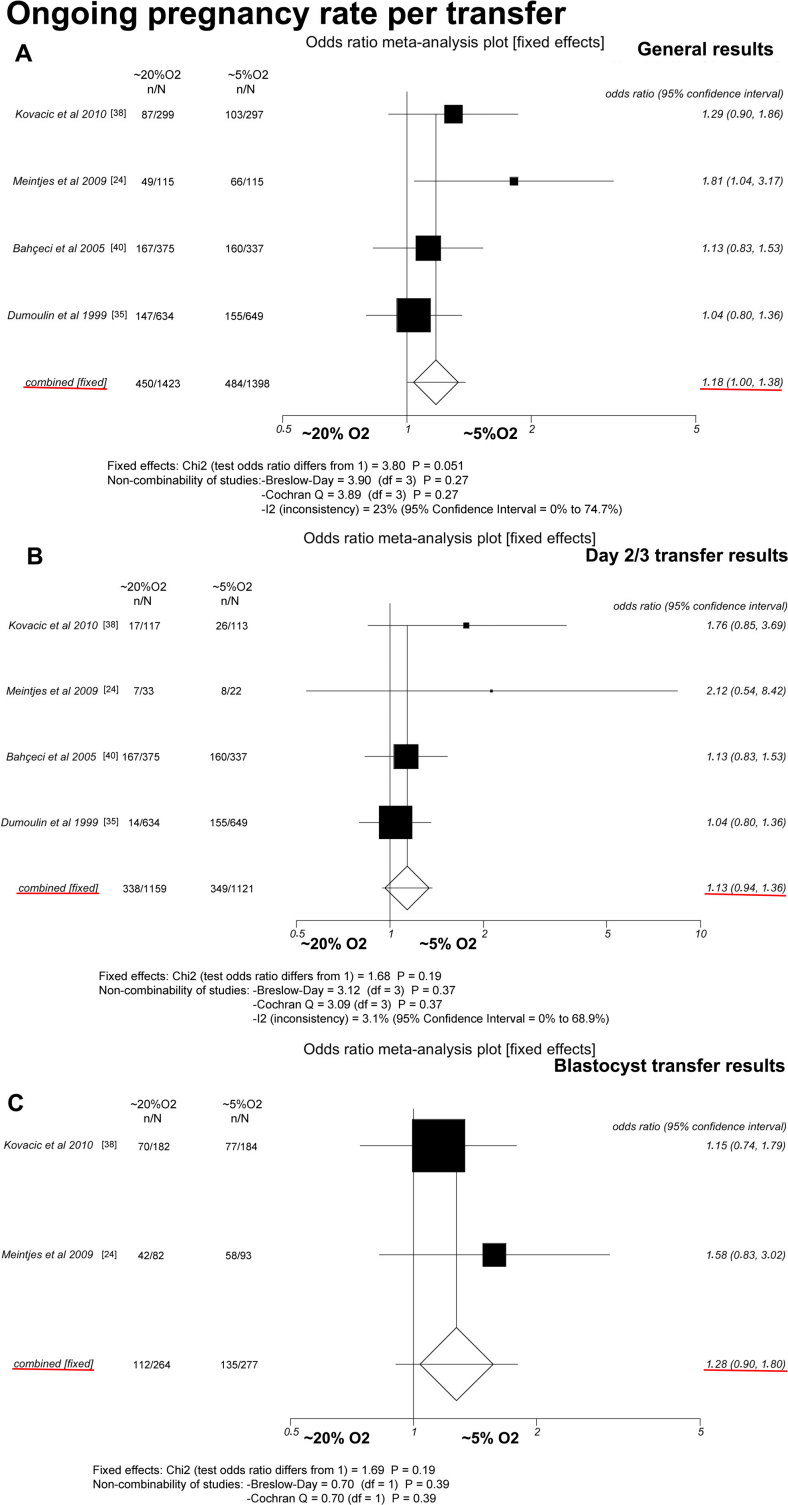
**Fixed-effect model**. Ongoing pregnancy rates. A: All cycles; B: only cycles in which embryos were transferred on day 2 or 3; C: only cycles in which embryos were transferred at the blastocyst stage.

Similarly, in the subgroup of cycles in which embryos were transferred on day 2 or 3 [24,35,38,40], the pooled OPR per transfer did not differ significantly between the group of patients that received sets with only OC~5 embryos (31.1%, 349/1, 121) and the group receiving sets with only OC~20 embryos (29.2%, 338/1, 159) (*P *= 0.19; OR = 1.13, 95% CI = 0.94-1.36) using a comparison without heterogeneity (Breslow-Day = 3.12, df = 3, P = 0.37; Cochran's Q = 3.09, df = 3, P = 0.37; I^2 ^= 3.1%, 95% CI = 0% to 68.9%).

Additionally, when a meta-analysis was performed only with cycles in which embryos were transferred at the blastocyst stage [24,38], the OPR per transfer was again not significantly different between the transferred sets with only OC~5 embryos (48.7%; 135/277) and the sets with only OC~20 embryos (42.4%, 112/264) (*P *= 0.19; OR = 1.28, 95% CI = 0.90-1.80). There was no heterogeneity in this comparison (Breslow-Day = 0.70, df = 1, *P *= 0.39; Cochran's Q = 0.70, df = 1, *P *= 0.39).

## Discussion

A review of the medical literature was necessary to absorb the increasing volume of published information. Meta-analysis seeks to answer a certain clinical question using a well-defined strategy to locate relevant evidence, assess the available studies using clear methodological criteria and formally summarise the results. This method consists of an analytical approach in which different, independent studies are integrated and the results are combined into a single common result. Compared with narrative reviews, meta-analysis has the great advantage of being less influenced by a reviewer's personal opinion, thus providing impartial conclusions. Additionally, all the results can easily be recalculated and compared with the conclusions stated by the authors. Even when it does not produce definitive conclusions about the usefulness of a treatment or technique, a meta-analysis may indicate the need for further randomised studies on the subject. Furthermore, previous meta-analyses allow the identification of the most important issues to be analysed in future research studies. Based on positive results in animals, several RCTs evaluating the effects of gamete and embryo cultures at low O_2 _concentrations (~ 5%) on clinical outcomes have been published, especially recently [24,28,35,36,38-40]; however, the conclusions of these studies have not always been consistent. Therefore, given the clinical potential of this method, a review of this subject was deemed useful.

Despite the apparent positive trend with low O_2 _levels, the results showed similar rates of fertilisation, implantation (generally and in the subgroup with transfer at day 2 or 3), and ongoing pregnancy. Hence, despite some positive results, it seems premature to recommend the use of low O_2 _tension for oocyte and embryo culture. To achieve a low oxygen concentration, an incubator should have sensors for both CO_2 _and O_2_; however, the commonly used units only have sensors for CO_2_. In addition, nitrogen is initially used to purge O_2 _from the incubator. Thus, the cost of supplying these three gases is greater than that for a typical incubator because each time the incubator is handled (i.e., when the door is opened), the interior atmosphere must be re-equilibrated with a further injection of nitrogen or a balanced mixture of gases to maintain a low O_2 _concentration [45]. One option would be exchanging the typical large incubators for low-volume units (e.g., benchtop incubators) that use continuous gas flow [46,47]. In either case, the decision to use low O_2 _concentrations implies necessary changes in laboratory practices with important economic repercussions. Therefore, we believe that additional randomised controlled trials are still necessary before evidence-based recommendations can be provided.

However, in the present meta-analysis, embryonic culture at low O_2 _concentrations displayed favourable effects The implantation rate was highly significant for blastocyst transfers only (P = 0.006). With only 4 studies evaluated for implantation rate and ongoing pregnancy rate, the *P*-values were 0.06 and 0.051 for these parameters. This being two of the three defined end-points for this study suggests that low-oxygen culture may be beneficial. Similar positive results excluded from this meta-analysis (e.g., data reported by only one study or data presented in a way making calcualtions impossible) have been reported by some RCTs (increases in biochemical [37,39] and cumulative [38] pregnancy rates and higher rates of live births [24]). In addition, other non-randomised studies have also reported increased implantation [21] and pregnancy [21,48,49] rates. On the basis of these data, it may seem attractive to consider low O_2 _tension as a means to improve clinical outcomes.

Meta-analysis also presents problems, such as the quality of the primary studies or the form in which data are reported and the dependence on a sufficient number of eligible studies to justify the statistical analysis. Methodological problems caused by clinical heterogeneity and insufficient power (low sample size) cause difficulty in drawing inferences from the meta-analysis. In this study, heterogeneity was found only for the general implantation rate, which may be related to the calculations being carried out with the results of transfer of embryos in the cleavaged stage and in the blastocyst stage together. No heterogeneity was found for the other outcomes. This reflects a relative agreement among the trials regarding the studied parameters and has particular importance when statistically significant outcomes were found. Nevertheless, it should be stressed that tests of heterogeneity among studies have low power. Therefore, where there is a non-significant test for heterogeneity among studies, it may be that a relationship is present but remains undetected.

This meta-analysis failed to show any statistically significant differences in the most clinically relevant outcome: the ongoing pregnancy rate. This observation may be related to the small cumulative sample size (i.e., insufficient power). With respect to the ongoing pregnancy rates, based on the results obtained with OC~5 and OC~20 embryo sets in the general population (34.6%, 31.6% and 484/1, 398, 450/1, 423, respectively), the subgroup with transfers at day 2 or 3 (31.1%, 29.2% and 349/1, 121, 338/1, 159, respectively) and the subgroup with transfers at the blastocyst stage (48.7%, 135/277 and 42.4%, 112/264, respectively), the ability to detect a difference of 5% with a power of 80% would require approximately 4000 (general population), > 4, 000 (day 2 or 3) and 2, 000 patients blastocyst) respectively, to reach definitive conclusions. Thus, for more consistent conclusions, this meta-analysis suggests that researchers should wait for the results of new RCTs that provide more information on clinical parameters. In addition, although smaller studies conducted on diverse populations may better reflect the natural heterogeneity of treatment effectiveness as found in daily practice, large studies may produce a more precise answer. Thus, further RCTs with larger sample size will be helpful for the corroboration of these results.

In many animal studies, the favourable effect of low oxygen concentration has been reported in the late stages of embryonic (the blastocyst stage) and even in foetal development [6,8-10,12,15,16,50-54]. For this reason, the necessity of reducing oxygen levels has been associated with blastocyst culture. In fact, more recent studies evaluating the transfer of human blastocysts have reported significantly better results with the use of low concentrations of oxygen (increased embryo quality [24,28,36,37] and higher implantation [24], pregnancy and live birth [24,37] rates). Unfortunately, the differences in embryonic evaluation criteria and in the method of presenting the data among these studies precluded the assessment of embryo quality in this meta-analysis. Although this meta-analysis identified higher implantation rates in the group of embryos cultured at ~5% O_2 _than those grown at atmospheric O_2 _levels with respect to blastocyst transfer, it failed to identify any difference in the rates of ongoing pregnancies. Thus, despite the studies suggesting a direct action on the results, the clinically relevant beneficial effects of low O_2 _concentration were not confirmed. However, it is important to note that we cannot rule out that these negative results may be related to the small number of studies that met the inclusion criteria (only 2), leading to a cumulative sample size insufficient to detect a significant difference. Again, future controlled trials will help to clarify this issue.

It has been argued that deleterious effects of high oxygen levels can have an effect on embryos during the early developmental stages despite the later onset of symptoms that occur during or subsequent to the blastocyst stage, which may affect clinical outcomes [6,24,46,50]. Recently, Nannasy et al. [55] reported that human embryos cultured at ~20% oxygen concentration for days 1-2 and subsequently at 5% oxygen for days 3-5 did not display better implantation, pregnancy, or blastulation rates than embryos maintained at 20% oxygen for the entire time period (0-3/5 days). The authors speculated that, among the possible explanations, embryos cultured in low oxygen on days 3-5 may not be able to overcome defects induced during days 1-2 of culture in atmospheric oxygen. Nonetheless, human studies examining O_2 _tensions throughout the culture period and performing embryo transfers at day 2 or 3 [21,24,35,38,40] did not identify, in most cases, beneficial clinical outcomes associated with reduced oxygen levels, and these data were confirmed by the calculations of this meta-analysis. Future controlled trials will help to clarify this issue and should include results from later clinical stages, such as live birth rate, in the analysis.

In addition, we should consider that several factors, such as differences in patient structure, embryo transfer policy, duration of cultivation, and culture media, could contribute to the observation of the contradictory outcomes of published studies on low oxygen. The possible more/less protective effect of various culture media against ROS and different concentrations of protective molecules has to be highlighted. Problems with the maintenance of stable low O_2 _atmosphere in the incubators during manipulation of embryos should also be stressed. Such differences must be considered in future trials or meta-analysis.

In conclusion, the findings of this meta-analysis demonstrate that, despite some promising results, it seems too early to conclude that low O_2 _culture conditions has an effect on IVF outcome. Additional randomised controlled trials are necessary before evidence-based recommendations can be provided. It should be emphasised that the present meta-analysis does not provide any evidence that low oxygen concentration is unnecessary.

## Conflicts of interests

The authors declare that they have no competing interests.

## Authors' contributions

DBGS designed and coordinated the study. All authors were responsible for the data collection, analysis, and interpretation presented in the manuscript. DBGS, JBAO, RLRB and JGF performed the statistical analyses and wrote the manuscript; JGF reviewed the manuscript. All authors read and approved the final manuscript.
